# Achieving myoblast engraftment into intact skeletal muscle via extracellular matrix

**DOI:** 10.3389/fcell.2024.1502332

**Published:** 2025-01-14

**Authors:** Kitora Dohi, Yasuko Manabe, Nobuharu L. Fujii, Yasuro Furuichi

**Affiliations:** Department of Health Promotion Sciences, Graduated School of Human Health Sciences, Tokyo Metropolitan University, Hachioji, Japan

**Keywords:** skeletal muscle, myoblast, muscle stem cell, cell transplantation, extracellular matrix

## Abstract

Cell therapy of skeletal muscles is a promising approach for the prevention of muscular diseases and age-related muscle atrophy. However, cell transplantation to treat muscle atrophy that does not involve disease, such as sarcopenia, is considered impossible because externally injected cells rarely engraft into non-injured muscle tissue. Additionally, skeletal muscle-specific somatic stem cells, called satellite cells, lose their ability to adhere to tissue after being cultured *in vitro* and transforming into myoblasts. To overcome these hurdles, we explored using extracellular matrix (ECM) components to create a niche environment conducive for myoblasts during transplantation. We demonstrated that myoblasts mixed with ECM components can be engrafted into intact skeletal muscle and significantly increase muscle mass in a mouse model. These findings implicate cell transplantation therapy as a viable option for the treatment of sarcopenia. The findings will inform advancements in regenerative medicine for skeletal muscles.

## 1 Introduction

Age-related skeletal muscle atrophy and weakness, known as sarcopenia, is a major problem, especially in developed countries. Sarcopenia not only impairs physical activity through muscle dysfunction but also reduces resistance to various diseases such as dementia, diabetes, and heart disease, ultimately reducing quality of life (Janssen and Ross, 2005; Rosenberg, 1997; Heitmann and Frederiksen, 2009). Resistance exercise with appropriate nutritional intake is the only way to maintain skeletal muscle mass, but for the elderly and physically disabled people with advanced sarcopenia, performing high-intensity exercises that restore muscle mass and strength carries the risk of accidents and disability. Currently, there is no effective drug for sarcopenia, and many researchers are trying to establish a method to treat and prevent age-related muscle atrophy.

Cell transplantation into skeletal muscle tissue is widely believed to potentially offer a rapid therapeutic intervention for muscle atrophy. Skeletal muscle, which has a high regenerative capacity, contains somatic stem cells called satellite cells. Satellite cells are located between the plasma membrane of muscle cells (myofiber) and the basal lamina; importantly, they have a high myogenic potential. Although they are normally quiescent in healthy adult muscle tissue, satellite cells are activated when myofibers are damaged and differentiate into myoblasts (Dumont et al., 2015; Yin et al., 2013). Myoblasts proliferate and fuse with each other to repair damaged myofibers or create new ones, while some return to a quiescent state to maintain a stem cell pool in preparation for the next injury (Dumont et al., 2015). Naturally, satellite cells and myoblasts can be expected to repair myofibers when they are transplanted into atrophied muscles (Hekmatnejad and Rudnicki, 2022; Mueller and Bloch, 2020). In fact, cell transplantation into muscle tissue is already in use as a treatment for genetic muscle diseases such as Duchene muscular dystrophy (DMD) (Hekmatnejad and Rudnicki, 2022; Mueller and Bloch, 2020; Bentzinger et al., 2014; Judson et al., 2018). However, muscle cell transplantation is considered a last resort for muscle therapy, limited to severe muscle disease, and has not been used to prevent the muscle atrophy that typically occurs with aging.

The reason why cell transplantation has been deemed unfeasible for the treatment of sarcopenia is that transplanted cells tend to survive only within injured skeletal muscle and are expected to die when transplanted into intact muscle tissue. In DMD muscle, myofibers collapse faster than they are repaired, causing muscle atrophy (Gutpell et al., 2015; Guiraud et al., 2019). Muscle regeneration is constantly occurring in damaged muscle, and the micro-environment (niche) is adapting to it. For example, the extracellular matrix (ECM) is reconstructed, and immune cells or fibro-adipogenic progenitors accelerate the activation of satellite cells and the proliferation and differentiation of myoblasts (Fuchs and Blau, 2020). The injury-induced regenerative niche is also suitable for engraftment of transplanted cells because the existing myofibers are destroyed and the regeneration program is initiated, allowing the transplanted cells to efficiently contribute to the newly regenerated muscle (Ikemoto et al., 2007; Azzag et al., 2022; Sacco et al., 2008). Conversely, in conditions like sarcopenia, where atrophied muscles do not undergo myofiber damage or regeneration, the transplanted cells cannot be incorporated into the regeneration process and are subsequently eliminated (Ikemoto et al., 2007; Azzag et al., 2022; Sacco et al., 2008). Many studies have already indicated that muscle injury by radiation or cardiotoxin (CTX) prior to cell transplantation, such as in DMD model animals, is an essential step for the engraftment of cell transplantation in muscle tissue (Hekmatnejad and Rudnicki, 2022; Mueller and Bloch, 2020). However, for human therapeutic applications, it is not acceptable to damage skeletal muscle for cell transplantation. We need to explore techniques that enable the engraftment of myoblasts into intact muscle tissue.

Another difficulty in realizing skeletal muscle cell therapy is that satellite cells have significantly reduced engraftment efficiency after being cultured as myoblasts. Although satellite cells have a higher engraftment efficiency than myoblasts (Ikemoto et al., 2007; Sacco et al., 2008; Montarras et al., 2005), they are inherently limited in number (Ono et al., 2010). Consequently, they require expansion *ex vivo* to reach quantities suitable for regenerative medicine applications. This is because skeletal muscle, as a large tissue distributed throughout the body, requires a substantial number of cells to significantly increase muscle mass by transplantation. However, cultured satellite cells promptly transition to myoblasts upon proliferation (Machado et al., 2017), reducing transplantation efficiency (Ikemoto et al., 2007; Sacco et al., 2008). This predicament highlights a critical gap in achieving successful skeletal muscle cell transplantation, requiring innovative solutions to realize the potential of regenerative medicine.

When culturing myoblasts, we must coat dishes with ECM components such as Matrigel (Chaturvedi et al., 2015; Penton et al., 2016; Thomas et al., 2015). Lacking the ECM coating, myoblasts cannot adhere to the dishes and fail to proliferate or differentiate. Therefore, we hypothesized that by replacing the niche environment with ECM components that induce myoblasts to proliferate and differentiate during the transplantation process, myoblasts could successfully engraft into intact muscle tissue. Here, we demonstrated that myoblasts mixed with Matrigel can engraft into intact skeletal muscles in a mouse model. We also studied the amount of Matrigel, the syringe used for transplantation, and the number of transplanted cells. On optimizing the procedure, myoblasts transplanted with Matrigel were highly efficient in engrafting into intact skeletal muscle and were successful in increasing muscle weight. Our study demonstrates the potential of cell transplantation in skeletal muscle regenerative medicine, with prospects for the treatment of sarcopenia.

## 2 Materials and methods

### 2.1 Animals

Adult C57BL/6 WT mice (8–16 weeks old, from Jackson Laboratory or Japan SLC) and adult CAG-EGFP mice (8–16 weeks old, from Japan SLC) were used as host and donor mice, respectively, for transplantation experiments. These mice were fed normal chow and water under standard lighting conditions (12 h light/dark cycle) at 23°C–25°C.

### 2.2 Isolation of single muscle fibers and primary myoblast culturing

The extensor digitorum longus (EDL) was isolated from CAG-EGFP mice, and single muscle fibers were digested using type I collagenase as previously described (Furuichi et al., 2021). For culturing satellite cells, the collagenase concentration was 0.25% (w/v), and the incubation time was 2 h at 37°C. Single muscle fibers were collected under a stereomicroscope; cells and debris other than muscle fibers were removed. The muscle fibers were pooled and incubated with Accutase (Innovative Cell Technologies, SAN, United States) for 10 min at room temperature and cultured on Matrigel-coated dishes. Myoblasts derived from satellite cells were cultured in growth medium (No glucose DMEM supplemented with 30% (v/v) FBS (NICHIREI BIOSCIENCES INC., Tokyo, Japan), 1% (v/v) GlutaMAX, 1% (v/v) chicken embryo extract, 10 ng/mL bFGF and 1% (v/v) penicillin–streptomycin) at 37°C with 5% CO_2_ (Furuichi et al., 2021). Myoblasts were expanded 7–8 days for the transplantation study.

### 2.3 Intramuscular cell transplantation

Myoblasts derived from green fluorescent protein (GFP) mice were removed from culturing dishes with 0.25% trypsin-EDTA (Gibco) and centrifuged at 1,000 rpm for 5 min before the supernatant was discarded. The cell pellet was then slowly suspended in growth medium, and a portion of the suspension was used for cell counting (OneCell, Japan). A sample (1.0 × 10^5^) of cells was removed from the cell suspension and centrifuged at 500 g for 5 min and mixed with Matrigel (final concentration was 1.0 × 10^5^ cells per 50 µL with 0 mg/mL, 0.5 mg/mL, 2.5 mg/mL, and 5.0 mg/mL Matrigel) or without Matrigel (only DMEM). The prepared cell suspension was injected into the tibialis anterior (TA) muscle of WT mice anesthetized with 7.5% Domitor, 8% midazolam, 10% butorphanol, and 74.5% saline (0.05 mL per 10 g mouse body weight). Anesthetized mice were shaved around the TA muscle. A 26 gauge (G) plastic syringe was injected from the bottom of the TA near the distal tendon to the top of the muscle and held for 10 s. The starting point of each needle injection was 2 mm above the TA distal tendon. The needle was pushed forward straight to the side of the knee within the TA muscle. The insertion of the needle was always stopped before the end of the muscle. The injection depth was approximately 500 µm deeper than the TA muscle surfaces. Then, 50 µL of the prepared cell suspension was injected over 1 min, and the syringe was removed. After confirming that the injected cell suspension did not leak out, the mice were awaked by an anti-anesthetic (5 mg/mL Antisedan in saline, ZENOAQ, Japan), using the same dose as what was used to anesthetize them. To determine the optimal needle and syringe, a 26 G plastic syringe (JMS Co., Ltd., Japan), a 27 G (TERUMO CORPORATION, Japan), a 29 G (Becton, Dickinson and Company, USA), or a 26 G glass syringe (Hamilton, GL Science, Japan) was used. For muscle injury, TA muscles of the anesthetized WT mice were injected with 100 µL of cardiotoxin at a concentration of 10 µM (Latoxan, France) 1 day prior to transplantation. Three or 6 weeks later, the transplanted TA muscles were dissected and embedded in Tissue-Tek O.C.T. compound (Sakura) to fix in isopentane cooled with liquid nitrogen. The frozen muscles were stored at −80°C until they were cryosectioned.

### 2.4 Mouse TA sampling, cryosectioning, staining, and imaging

The frozen TA muscles were sectioned (8–10 µm) using a cryostat (Leica). The sections were immediately observed to acquire their GFP signal using a KEYENCE BZ-X710/810. For immunostaining, the sections were fixed in 4% paraformaldehyde or acetone for 10 min and blocked with 20% goat serum in PBS for 30 min at room temperature. The sections were then incubated with primary antibody (Laminin-α2; 1:100, Enzo Life Science) in blocking buffer at 4°C overnight before being washed with PBS and labeled with fluorescence-conjugated secondary antibody (Alexa594-anti Rat, Invitrogen) for 1 h at room temperature. The sections were mounted using mounting medium with DAPI (Vector Laboratory). Laminin and DAPI were visualized using a KEYENCE BZ-X710/810, and the images were merged with the GFP images (Supplementary Figure S1). The merged images were analyzed by ImageJ (for counting GFP positive fibers) or KEYENCE BZ-X series Image Analysis Software (for detecting GFP positive area, GFP fluorescence intensity, and fibrosis area).

To stain the α-Bungarotoxin protein, the sections were blocked with 3% BSA, 5% goat serum, and 0.1% Triton X-100 in PBS and incubated with Laminin-α2 primary antibody. The following day, the sections were reacted with α-Bungarotoxin fluorescence-conjugated antibody (1:500, Alexa594, Invitrogen) and Laminin-α2 secondary antibody for 2 h at room temperature and mounted with DAPI.

To stain the neurofilament protein, the sections were blocked with the same solution of α-Bungarotoxin staining and incubated with ready-to-use anti-Neurofilament (Dako) at 4°C overnight. After washing to remove excess unbound primary antibody, the sections were reacted with fluorescence-conjugated secondary antibody (1:500, Alexa594-anti-Mouse, Invitrogen) for 2 h at room temperature and mounted with DAPI.

CD31 staining, the sections were fixed in acetone for 10 min at −20°C and blocked with 5% BSA, 1% goat serum, and 0.05% Triton X-100 in PBS for 1 h at room temperature. The sections were then incubated with primary antibody (CD31; 60 ng, BioLegend) in blocking buffer for 2 h at room temperature. After washing to remove excess unbound primary antibody, fluorescence-conjugated secondary antibody (1:200, Alexa594-anti-Rat, Invitrogen) was added for 2 h at room temperature, followed by mounting with DAPI.

For hematoxylin and eosin (HE) staining, muscle cryosections were washed in PBS for 3 min and stained in hematoxylin solution (Cosmo Bio, Tokyo, Japan) for 4 min. The sections were washed in running water for 1 min and stained in eosin solution (Cosmo Bio, Tokyo, Japan) for 5 min before being washed in distilled water for 2 min. The sections were incubated in 70%, 80%, 90%, and 100% ethanol for 1 min, 2 min, 3 min, and 5 min and then mounted with a hydrophobic mounting medium.

For Sirius Red staining, muscle cryosections were fixed in 4% PFA for 10 min and washed twice in 100% ethanol for 5 min and 15 min. After washing, the sections were dried for 30 min, washed in distilled water for 1 min, and incubated in Sirius Red solution (1.3% picric acid; Wako, Osaka, Japan, 0.1% Direct Red80; Sigma, St. Louis, USA) for 45 min. The sections were washed in 0.5% acetic acid for 5 min and distilled water for 1 min before being mounted with a hydrophobic mounting medium. The percentage of collagen area in the whole TA (%) was evaluated using the formula {collagen-positive area (µm^2^)/whole TA area (µm^2^)} × 100. The region of interest (ROI) was defined as the square that enclosed all GFP + fibers within the cross section, where each corner of the square was set to coincide with the outermost GFP fibers (Supplementary Figure S2). The collagen area in the ROI (%) is evaluated using the formula {collagen-positive area in ROI (µm^2^)/TA area in ROI (µm^2^)} × 100.

### 2.5 RNA isolation and real-time quantitative PCR analysis

Total RNA was extracted from skeletal muscle tissue using TRIzol reagent (Invitrogen). RNA was transcribed into cDNA via a standard reverse transcriptase reaction using the PrimeScript^TM^ first-strand cDNA Synthesis Kit (Takara, Shiga, Japan), following the manufacturer’s protocol. These cDNAs were analyzed with quantitative real-time PCR (qPCR). qPCR was performed on a 96-well CFX Connect Real-Time PCR Detection System (Bio-Rad Laboratory, Tokyo, Japan) using a THUNDER-BIRD Probe qPCR Mix (TOYOBO, Osaka, Japan). The mRNA levels of GFP or TNF-α were normalized to that of the housekeeping gene TATA-binding protein (Tbp). Primer sequences 5′-3′ for this study were as follows: GFP (forward; ACG​TAA​ACG​GCC​ACA​AGT, reverse; AAG​TCG​TGC​TGC​TTC​ATG​TG); TNF-α (forward; ATGGGGGGCTTCCAGAA, reverse; CCT​TTG​GGG​ACC​GAT​CA); Tbp (forward; AAT​GAC​TCC​TAT​GAC​CCC​TAT​CAC, reverse; AGG​TCA​AGT​TTA​CAG​CCA​AGA​TTC). The mRNA content of both GFP and Tbp was calculated from the cycle threshold values using a standard curve, and the ratio between GFP and Tbp was calculated. Primers were synthesized by Eurofins Genomics Co., Ltd. (Tokyo, Japan).

### 2.6 Measurement of contractile force TA muscles *in vivo*

Under anesthesia, as described above, the TA muscle was exposed and isolated at its distal tendon. The mouse was placed on the mouse muscle tension measurement system (Uchida Denshi, Tokyo, Japan), and the mouse knee was fixed with forceps. The distal TA tendon was attached to a force transducer, and a pair of platinum-coated electrodes was placed on the common peroneal nerve for electrical stimulation with rectangular unipolar 1.0 voltage pulses of 0.2 m duration at a frequency of 250 Hz. The TA muscle was maintained at optimal length, and the maximal tetanic force was determined from stimulation with the above signal for 10 s. The maximum voltage in the first contraction was determined as the contraction force (Supplementary Figure S3).

### 2.7 Statistical analyses

All values are shown as the mean ± standard error (s.e.m.). Two-tailed unpaired Student’s t-tests were used to compare the two groups. For multiple comparisons, data were analyzed using a one-way ANOVA or two-way ANOVA followed by the Tukey *post hoc* test or Dunnett’s test. The significance was set to *p*< 0.05. Sample numbers *n* in all experiments were independent replicates.

## 3 Results

### 3.1 Matrigel enhanced the engraftment of transplanted myoblasts into intact muscle tissue

**FIGURE 1:**
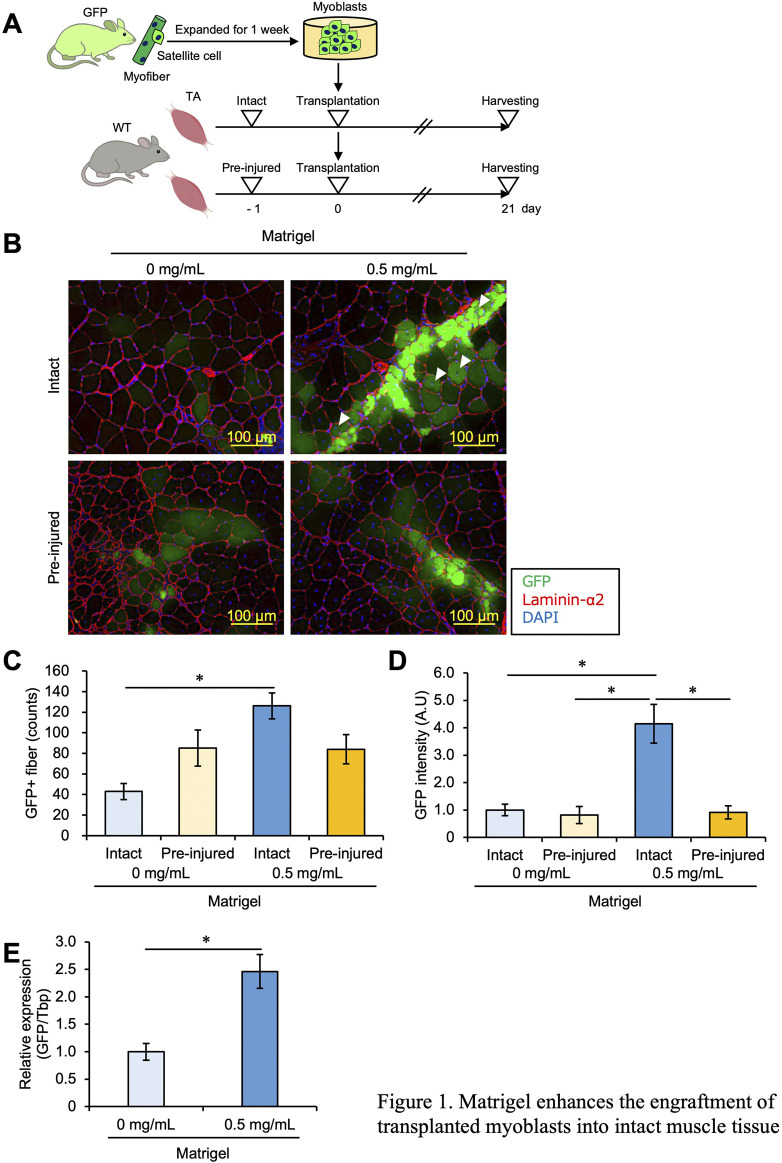
Matrigel enhances the engraftment of transplanted myoblasts into intact muscle tissue. **(A)** Experimental design of the myoblast transplantation study. Satellite cells were isolated from GFP mice and cultured for 1 week. Expanded myoblasts were then transplanted with a 26-G plastic syringe into the TA muscle of WT mice. Unilateral muscle injury was induced in host mice 24 h prior to cell transplantation. Twenty-one days post-transplantation, the TA muscles were dissected and analyzed. **(B)** GFP images were obtained from frozen sections of the transplanted TA muscle. The sections were stained with anti-Laminin-α2 (red) and DAPI for cell nuclei (blue). Scale bars are 100 µm. White arrows indicate immature GFP + myofibers with centered nuclei. **(C)** The number of GFP + fibers was quantified from the images in panel **(B)**. Data are shown as the mean ± s.e.m. (n = 8–10). Statistical significance was determined by a two-way ANOVA with Tukey’s honestly significant difference (HSD) test, with factor Matrigel *p* < 0.01 and interaction *p* < 0.01. **(D)** The intensity of GFP signals was detected and quantified from images in panel **(B)**. Data are shown as the mean ± s.e.m. (n = 8–10). Statistical significance was determined by a two-way ANOVA with a non-parametric Tukey test, with factors injury and Matrigel *p* < 0.001 and interaction *p* < 0.01. **(E)** GFP mRNA expression level of the intact muscles with or without Matrigel was quantified by q-PCR. Data are shown as the mean ± s.e.m. (n = 6–7). Statistical significance was determined by Student’s t-test.

In tissue, cells are covered by an ECM that preserves their shape and function; the ECM allows myoblasts to bind to the dish or each other for myogenesis, including proliferation and differentiation (Chaturvedi et al., 2015; Penton et al., 2016; Thomas et al., 2015). Myoblasts derived from GFP mice and cultured for 1 week were detached from the culture dishes using trypsin for transplantation. The myoblasts were separated into aliquots of 1.0 × 10^5^ cells and mixed with Matrigel, an ECM component, at a concentration of 0.5 mg/mL. We transplanted myoblasts into the TA muscle of WT mice with or without Matrigel. We also compared what happened when cells in either condition were transplanted into intact muscle or muscle with a CTX-induced injury. Twenty-one days later, the transplanted TA muscle was dissected and analyzed (Figure 1A).

As reported in previous studies, GFP + myofibers were observed in small numbers in pre-injured muscles but rarely in intact muscles, suggesting that myoblasts did not engraft into intact muscle (Figure 1B). However, the number of GFP + myofibers was increased when myoblasts mixed with 0.5 mg/mL Matrigel were transplanted into intact muscle as well as under pre-injury conditions (Figures 1B, C), indicating that ECM components such as Matrigel allowed myoblasts to engraft into muscle without injury. Moreover, when myoblasts mixed with 0.5 mg/mL Matrigel were implanted into intact muscle, GFP + myofibers with central nuclei, considered immature, were observed. Such immature GFP + myofibers were brighter than matured cells (Figure 1B). Note that in intact muscle, the number of GFP + myofibers was increased by adding Matrigel (Figures 1B,C); meanwhile, Matrigel had no effect on myoblast engraftment when the muscle was pre-injured (Figures 1B, C). In general, the number of GFP + fibers is interpreted as reflecting the engraftment efficiency of transplanted cells, but this could be an underestimate if the transplanted cells cluster together to form a single myofiber. Therefore, we measured the intensity of the GFP signal, which reflects the amount of GFP myoblasts, and found that the GFP intensity was significantly stronger when transplantation was carried out with Matrigel in intact muscle than in other conditions (Figure 1D). To validate the accuracy of our measurement of the amount of successfully fused GFP myoblasts, we measured the GFP mRNA expression level in intact conditions by qPCR and confirmed that the expression of GFP mRNA was higher with Matrigel than without Matrigel (Figure 1E). From these results, we concluded that ECM components enhanced myoblast engraftment into intact muscle and that this effect was specific to the intact condition.

Transplantation of allogeneic cells or injection of Matrigel may induce an immune response in host tissues. To evaluate an immune response caused by myoblast transplantation, we measured the expression of TNF-α mRNA, which is a well-known immune marker, in skeletal muscles. We confirmed that muscle injury induced by CTX injection increased TNF-α mRNA expression compared to basal control (Supplementary Figures S4A, B). Our data showed that TNF-α expression level was not increased with myoblast injection compared to injection of only saline, suggesting that the transplantation of myoblasts mixed with Matrigel had little effect on the immune response (Supplementary Figures S4C, D).

### 3.2 26 G plastic syringes were suitable for myoblast transplantation

Because cells were damaged as they passed through the syringe needle, we examined whether the syringe used for cell injection affected engraftment efficiency. Previously, many types of syringes were used for muscle cell transplantation; materials were mainly plastic or glass, and the needle diameters were different among researchers (Lorant et al., 2018; Arpke and Kyba, 2016; Hashimoto et al., 2004; Elhussieny et al., 2021; Parker and Tapscott, 2013; Motohashi et al., 2014; Praud et al., 2018). For example, glass can have a charge on the surface (Behrens and Grier, 2001), and this feature could affect cell viability, but it was unclear which materials were useful for myoblast transplantation. Moreover, the size of myoblasts was larger than that of satellite cells, so they may be more damaged by passage through a needle than satellite cells.

**FIGURE 2:**
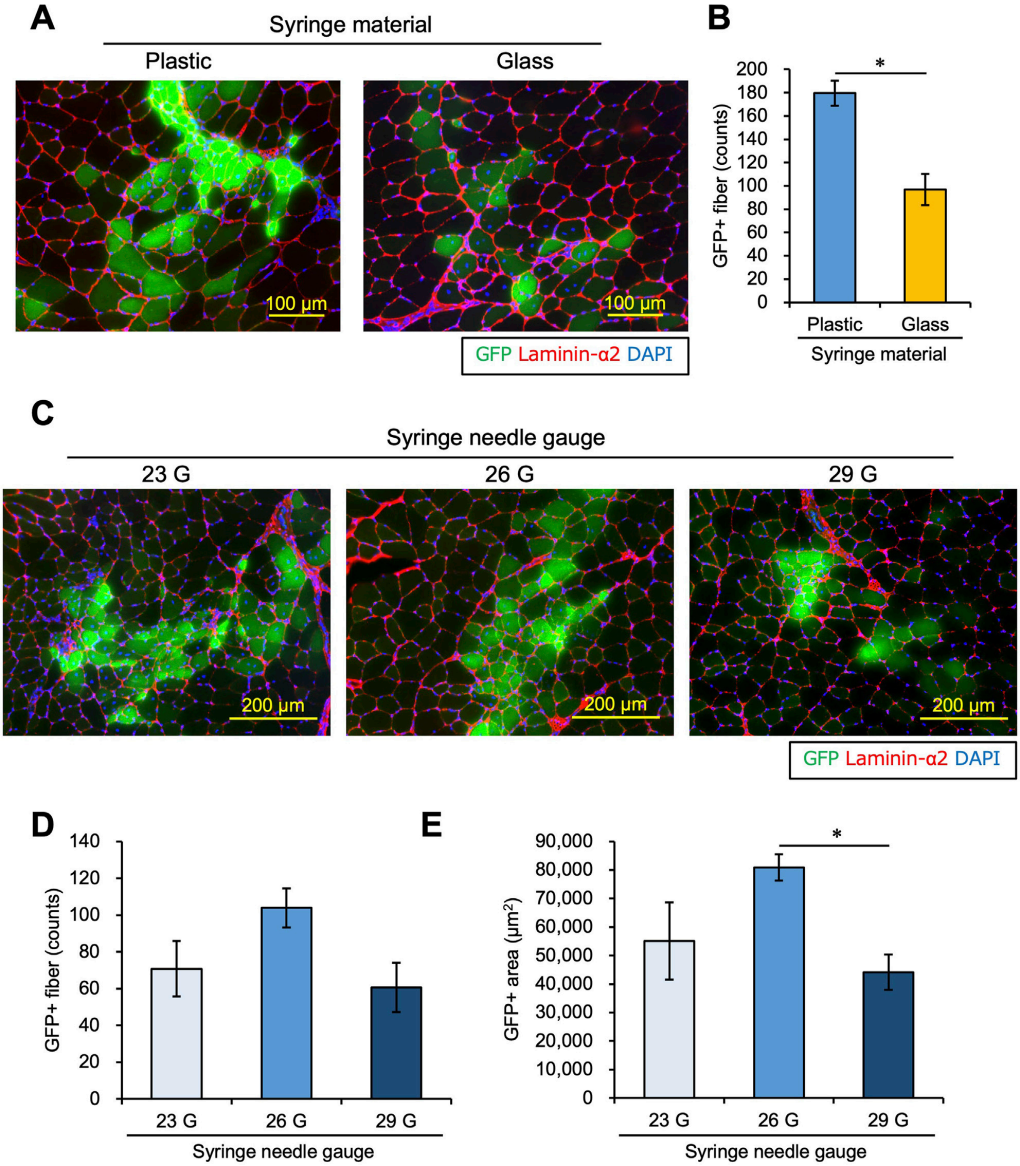
Syringe materials and needles affect the engraftment efficiency of transplanted myoblasts. **(A)** Myoblasts were transplanted into intact TA muscle with 0.5 mg/mL Matrigel using a plastic or glass syringe. Twenty-one days after transplantation, GFP images were captured using transplanted TA muscle sections. Sections were also stained with anti-Laminin-α2 (red) and DAPI (blue). Scale bars are 100 µm. **(B)** The number of GFP-positive myofibers was counted using the images in panel **(A)**. Data are shown as the mean ± s.e.m. (n = 6–7). Statistical significance was determined by Student’s t-test. **(C)** Using syringes with 23 G, 26 G, or 29 G needles, myoblasts were transplanted into intact TA muscle with 0.5 mg/mL Matrigel. Twenty-one days later, frozen sections of transplanted muscle were prepared. After capturing GFP (green) images, the sections were stained with anti-Laminin-α2 (red) and DAPI (blue). Scale bars are 200 µm. **(D)** The number of GFP-positive myofibers was counted from the images in panel **(C)**. Data are shown as the mean ± s.e.m. (n = 8). Statistical significance was determined by a one-way ANOVA, with *p* = 0.074. **(E)** The GFP-positive areas were measured from the images in panel **(C)**. Data are shown as the mean ± s.e.m. (n = 6–7). Statistical significance was determined by a one-way ANOVA with a non-parametric Tukey test.

First, we investigated whether plastic or glass is more suitable for transplanting myoblasts. We used plastic or glass syringes and transplanted 1.0 × 10^5^ myoblasts into intact TA muscle with 0.5 mg/mL Matrigel following the same schedule shown in Figure 1A. The number of GFP + myofibers was significantly reduced when a glass syringe was used (Figures 2A,B), suggesting that a plastic syringe is better for myoblast injection. This is supported by previous studies demonstrating that cells can adhere to glass cannulas (Torres et al., 2015; Amer et al., 2017), which may reduce the number of cells that completely transit a glass syringe.

Skuk et al. (2014) studied the relationship between the needle size and the engraftment efficiency of transplanted myoblasts in monkey models. The authors reported that a 27 G needle provided the best balance between minimizing host muscle injury and preserving the viability of donor-transplanted cells. However, in the mouse model, the optimal needle size is unclear. We explored the best needle size for myoblast transplantation using different needles, including 23 G, 26 G, and 29 G. We evaluated transplant efficiency after 21 days in the same way as in Figure 1. There was a trend of a higher number of GFP + fibers using a 26 G needle. However, the difference among the groups was not significantly different (one-way ANOVA *p* = 0.074, Figures 2C, D). On the other hand, the GFP + area was higher using a 26 G needle than with a 29 G needle, but there was no significant difference between 26 G and 23 G (Figure 2E). Therefore, the 26 G plastic syringe was deemed suitable for myoblast transplantation into intact muscle.

### 3.3 Fibrosis area increased with a high Matrigel concentration

**FIGURE 3:**
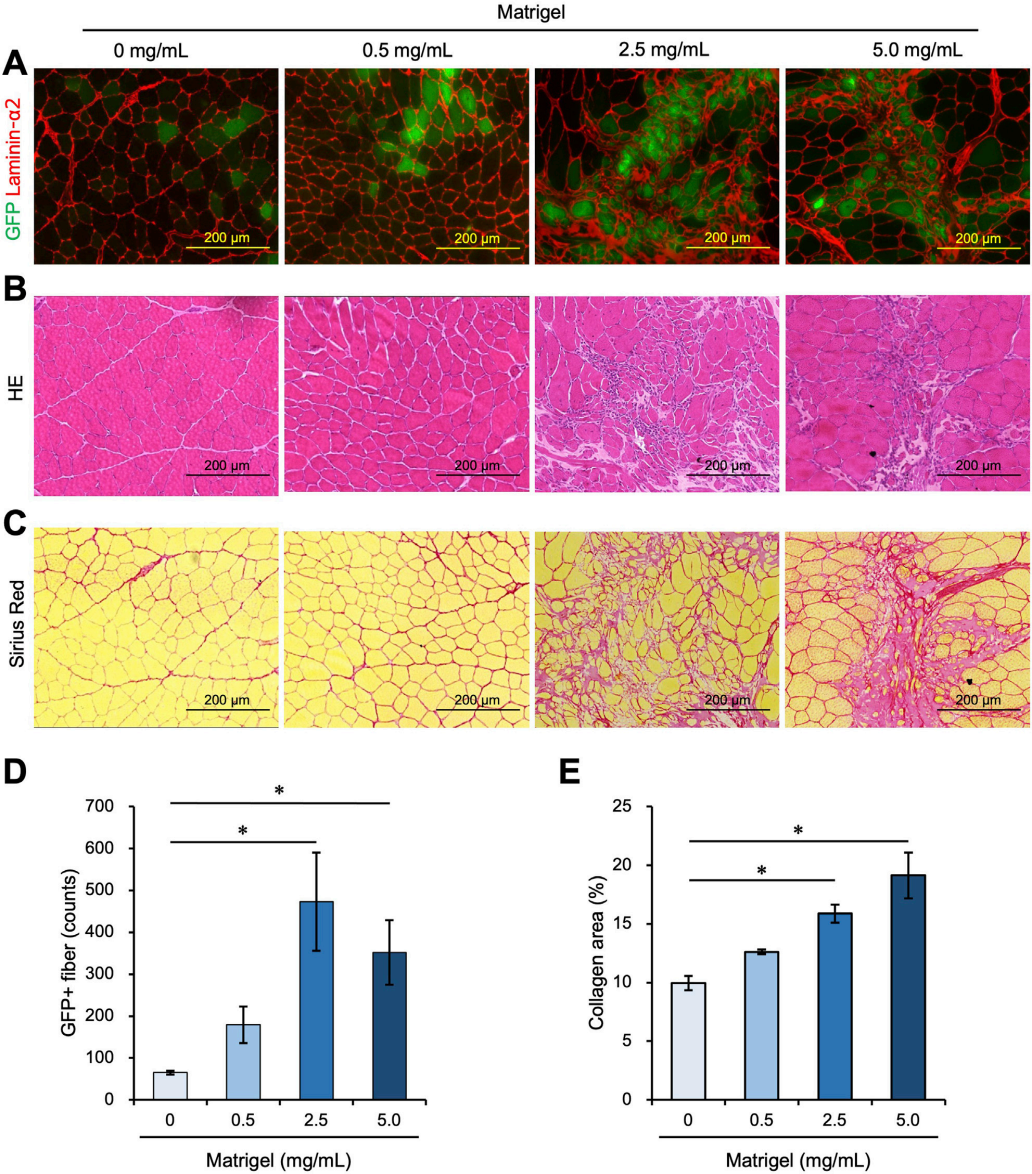
A high concentration of Matrigel enhances myoblast engraftment but causes fibrosis. **(A–C)** Frozen sections were prepared from TA muscle 21 days after myoblast transplantation mixed with 0 mg/mL, 0.5 mg/mL, 2.5 mg/mL, and 5.0 mg/mL Matrigel. Scale bars are 200 µm. **(A)** After capturing GFP (green) images, the sections were stained with anti-Laminin-α2 (red). **(B)** Frozen sections were stained with hematoxylin and eosin (HE). **(C)** Sirius Red staining of transplanted TA muscle, with myofiber stained in yellow and collagen stained in red. **(D)** The number of GFP-positive fibers was counted from the images in panel **(A)**. Data are shown as the mean ± s.e.m. (n = 4–5). Statistical significance was determined by a one-way ANOVA with a non-parametric Tukey test. **(E)** The percentage of collagen-positive area was quantified as fibrotic areas using the images in panel **(C)**. Data are shown as the mean ± s.e.m. (n = 4–5). Statistical significance was determined by a one-way ANOVA with a non-parametric Tukey test.

We examined whether further increasing the concentration of Matrigel would enhance the efficiency of myoblast engraftment into intact muscle. We transplanted 1.0 × 10^5^ myoblasts into intact TA muscle mixed with 0 mg/mL, 0.5 mg/mL, 2.5 mg/mL, and 5.0 mg/mL Matrigel. Twenty-one days after transplantation, the TA muscle was dissected and analyzed. The number of GFP + myofibers was higher in the 2.5 mg/mL and 5.0 mg/mL Matrigel concentrations than in the 0 mg/mL sample (Figures 3A,D). However, these high concentration conditions increased the non-myofiber area that was Laminin-α2 positive (Figure 3A). We prepared the cross section to confirm the morphology with HE staining and observed that the non-myofiber area showed signs of fibrosis (Figure 3B). Next, the transplanted muscle sections were stained with Sirius Red, which stains collagen in red and myofiber in yellow. This experiment revealed that the Laminin-α2 positive area matched the collagen-positive area, indicating that fibrosis occurred with the 2.5 mg/mL and 5.0 mg/mL Matrigel injections but not with the 0 mg/mL and 0.5 mg/mL injections (Figures 3A–C, E). Although myoblast transplantation with more Matrigel (especially 2.5 mg/mL) showed high engraftment efficiency, 2.5 mg/mL Matrigel caused fibrosis. If the fibrosis caused by the Matrigel injection could be eliminated, myoblast transplantation with 2.5 mg/mL Matrigel would be a better method to achieve high engraftment efficiency. We also examined the effect of injecting a high concentration (2.5 mg/mL) of Matrigel on the immune response (Supplementary Figures S4E, F). The expression of TNF-α was not changed following the injection of saline and 2.5 mg/mL Matrigel (Supplementary Figure S4F).

### 3.4 Increase in the number of transplanted myoblasts resulted in effective engraftment and suppressed fibrosis

**FIGURE 4:**
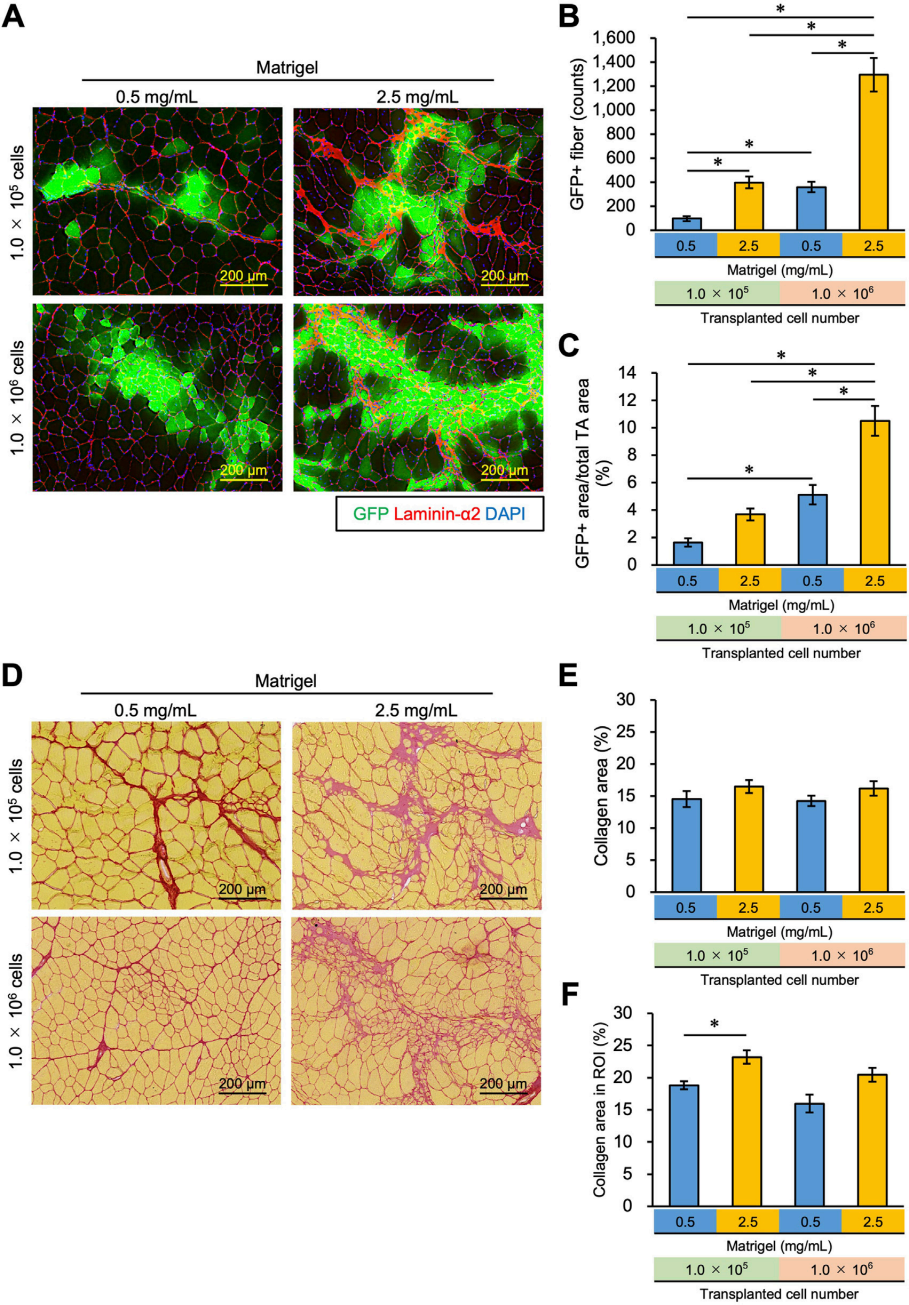
Increasing the concentration of Matrigel and transplanted myoblasts enhances transplantation efficiency and suppresses collagen deposition. **(A)** 1.0 × 10^5^ or 1.0 × 10^6^ myoblasts were mixed with 0.5 mg/mL or 2.5 mg/mL Matrigel and then transplanted into the TA muscle of WT mice. Frozen sections were prepared from TA muscle 21 days after myoblast transplantation. After capturing GFP (green) images, the sections were stained with anti-Laminin-α2 (red) and DAPI (blue). Scale bars are 200 µm. **(B)** The number of GFP-positive fibers was counted from the images in panel **(A)**. Data are shown as the mean ± s.e.m. (n = 5–7). Statistical significance was determined by a two-way ANOVA with a non-parametric Tukey test, with factors Matrigel and cell number *p* < 0.05 and interaction *p* < 0.05. **(C)** GFP + area was also measured from the images in panel **(A)**. Data are shown as the mean ± s.e.m. (n = 5–7). Statistical significance was determined by a two-way ANOVA with Tukey’s HSD test, with factors Matrigel and cell number *p* < 0.01 and interaction *p* < 0.05. **(D)** Sirius Red staining of the same sample in **(A)**. Myofibers were stained yellow; collagen was stained red. Scale bars are 200 µm. **(E)** The collagen area (%) in the whole TA muscle was measured from the images in panel **(D)**. Data are shown as the mean ± s.e.m. (n = 5–7). **(F)** After defining the region of interest (ROI), the collagen area (%) in the ROI was measured from the same images in panel **(D)**. ROI was defined as the rectangle that surrounded all GFP + fibers in the section. Data are shown as the mean ± s.e.m. (n = 5–7). Two-way ANOVA showed the main effects of cell number and Matrigel concentration without an interaction. The asterisk indicates a significant difference by Dunnett’s test compared to the control (0.5 mg/mL - 1.0 × 10^5^ myoblasts).

We examined whether increasing the number of transplanted cells from 1.0 × 10^5^ to 1.0 × 10^6^ could reduce the collagen area by stacking the transplanted cells. To do this, 1.0 × 10^5^ cells, and 10 times that number, 1.0 × 10^6^ cells, were mixed with 0.5 mg/mL or 2.5 mg/mL Matrigel and transplanted into TA muscle. We found that the number of GFP + fibers was observed to exceed 1,000 fibers when 1.0 × 10^6^ cells were transplanted with 2.5 mg/mL Matrigel, which was 10 times higher than that of the 1.0 × 10^5^ cells transplanted with 0.5 mg/mL Matrigel (Figures 4A, B). The GFP + area using 1.0 × 10^6^ cells in 2.5 mg/mL Matrigel was higher than in the other conditions, occupying almost 10% of the total TA area (Figures 4A, C; Supplementary Figure S1). The number and area of GFP + fibers were also found to increase with more transplanted cells (Figures 4A, C). We also analyzed the longitudinal cryosections, and they showed that the transplanted myoblasts were extensively engrafted in whole TA muscle, and the amount of the engrafted cells was slightly sparse in different locations (Supplementary Figures S5A, B). The GFP + fibers were formed in the same orientation as existing myofibers.

As shown in Figure 3, high-concentration Matrigel (2.5 mg/mL) increased the area where fibrosis was seen despite the increase in the engraftment of transplanted cells (Figure 4D). By transplanting 10 times as many cells (1.0 × 10^6^ cells), however, cells could invade the collagen area, suppressing the collagen deposition (Figure 4D). We quantified the collagen area over the whole TA cross section but failed to detect a significant difference between the conditions (Figure 4E). However, this analysis underestimates the change in collagen area due to Matrigel injection because it includes intrinsic collagen that is scattered throughout the muscle sections, even where myoblasts and Matrigel have not spread. Thus, we determined a region of interest (ROI), defining it as the area over which injected myoblasts and Matrigel have infiltrated and recalculated the collagen area in the ROI, as described in the Methods section (Supplementary Figure S2). Although the collagen area in the ROI increased when 1.0 × 10^5^ myoblasts were transplanted with 2.5 mg/mL Matrigel compared to 0.5 mg/mL (Figure 4F), there was no significant difference between 1.0 × 10^5^ myoblasts mixed with 0.5 mg/mL Matrigel and 1.0 × 10^6^ myoblasts transplanted with 2.5 mg/mL. Although no significant difference was observed by increasing cell number in the condition of 2.5 mg/mL Matrigel, our data imply that the increasing number of transplanted cells prevented the collagen accumulation caused by injecting high concentrations of Matrigel. We propose that 1.0 × 10^6^ myoblasts transplanted with 2.5 mg/mL Matrigel will engraft efficiently and will suppress fibrosis.

We also analyzed results 6 weeks after myoblast transplantation, which was twice the duration of the previous experiment. We transplanted 1.0 × 10^5^ or 5.0 × 10^5^ myoblasts mixed with 0.5 mg/mL or 2.5 mg/mL Matrigel into intact TA muscle and successfully detected the engraftment of myoblasts in all conditions (Supplementary Figure S6). When 1.0 × 10^5^ transplanted myoblasts were mixed with 0.5 mg/mL Matrigel, we observed 288.0 GFP + fibers, which was three times more than the count of 96.0 in the same conditions at 3 weeks (Supplementary Figure S6C, Figure 4B). Additionally, the area of GFP + as a percentage of the total TA area was also three times higher at 6 weeks than at 3 weeks (4.95% vs. 1.64%) (Supplementary Figure S6D, Figure 4C). Although Pavlath et al. (1994) reported that engrafted myofibers were reduced depending on the periods after injection, our data suggest that the engrafted myofibers can survive at least 6 weeks after transplantation.

### 3.5 Effect of myoblast transplantation on muscle weight and strength

**FIGURE 5:**
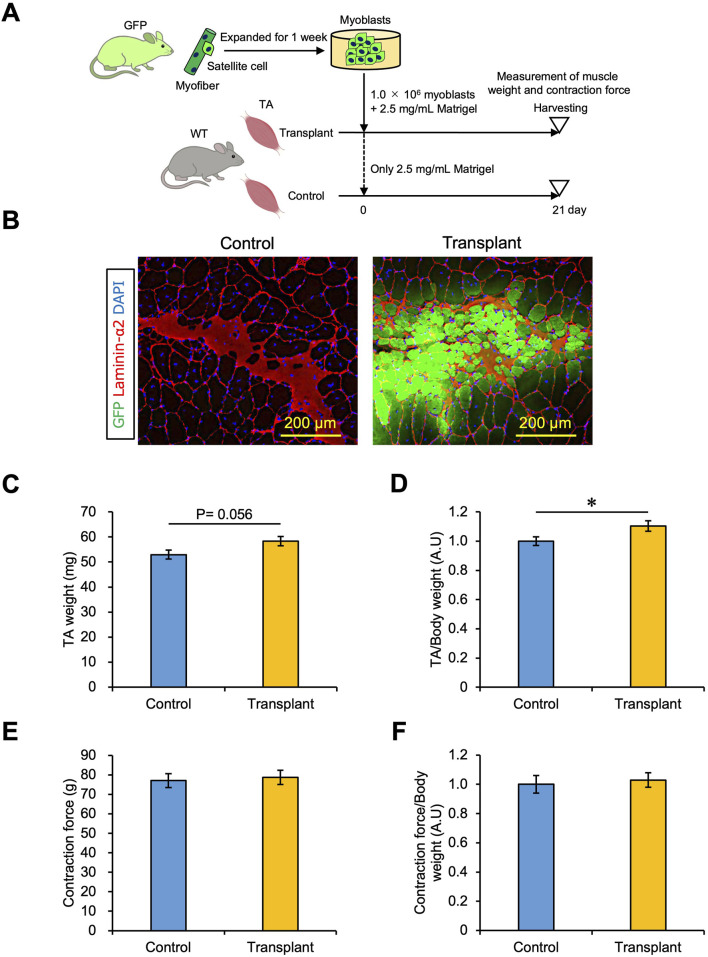
Effect of myoblast transplantation on muscle weight and strength. **(A)** Experimental schedule. 1.0 × 10^6^ myoblasts mixed with 2.5 mg/mL Matrigel were transplanted. Twenty-one days later, the muscle weight and strength were measured before harvesting. **(B)** GFP images were obtained from the frozen sections of transplanted TA muscle. The sections were stained with anti-Laminin-α2 (red) and DAPI (blue). Scale bars are 200 µm. **(C)** TA muscle weight was compared between control (only 2.5 mg/mL Matrigel) and transplant (1.0 × 10^6^ myoblasts mixed with 2.5 mg/mL Matrigel) sections. Data are shown as the mean ± s.e.m. (n = 9). Statistical significance was determined by Student’s t-test. **(D)** Transplanted TA muscle weight in **(C)** was normalized by body weight. Data are shown as the mean ± s.e.m. (n = 9). Statistical significance was determined by Student’s t-test. **(E)** Transplanted TA muscle strength was measured as the contraction force *in vivo*. An anesthetized mouse was placed on the mouse muscle tension measurement system, and the distal tendons of the tibialis anterior muscles were attached to a transducer. The TA muscle was contracted by electric stimulation, and the contraction force was detected. Data are shown as the mean ± s.e.m. (n = 7–9). **(F)** Transplanted TA muscle strength in **(E)** was normalized by body weight. Data are shown as the mean ± s.e.m. (n = 7–9).

As shown in Figure 4B, 1.0 × 10^6^ myoblasts transplanted with 2.5 mg/mL Matrigel generated more than 1,000 myofibers in TA muscle. We examined whether muscle weight and strength could be increased by myoblast transplantation in this manner. A sample of 1.0 × 10^6^ cells of myoblasts isolated from a GFP mouse was mixed with 2.5 mg/mL Matrigel and transplanted into one intact TA muscle of a WT mouse (shown as the Transplant condition), while the other TA was injected with only 2.5 mg/mL Matrigel as a control (Figure 5A). After transplantation, the TA with transplanted cells showed GFP + fibers (Figure 5B) and tended to increase the weight of the TA muscle (Figure 5C). Furthermore, the TA muscle weight normalized by body weight was increased by myoblast transplantation (Figure 5D). We demonstrated that the transplantation of myoblasts into intact muscle tissue could increase muscle weight by 10%. We also measured the strength of the transplanted TA muscle using the mouse muscle strength measurement system (Supplementary Figure S3). We found that the TA contraction force was not changed by myoblast transplantation, and the force normalized to body weight was the same (Figures 5E, F). Staining for α-Bungarotoxin and Neurofilament, which are expressed in the synapse structure, revealed that both markers were present in the GFP + fibers sample (Supplementary Figure S7). Moreover, we observed that CD31 expressed in endothelial cells was colocalized with GFP + fibers, suggesting that the vascularization occurred in the transplantation area (Supplementary Figure S8). Thus, our myoblast transplantation method did not show augmentation of muscle strength even if myofibers formed by transplanted cells had synapses and capillaries.

## 4 Discussion

Cultured myoblasts are not engrafted when transplanted into intact skeletal muscles, leading many to believe that this was not a viable strategy for cell transplantation therapies. In this work, we found that this prohibitive problem could be solved: myoblasts mixed with ECM components could indeed engraft into intact skeletal muscle tissue and even induce an increase in muscle weight in mice. Under optimal conditions, GFP + fibers were observed to exceed 1,000 counts, while GFP + area occupied 10% of the TA muscle. This constitutes significantly effective transplantation compared with previous studies (Ikemoto et al., 2007; Sacco et al., 2008; Montarras et al., 2005), while the increase in muscle weight by transplantation demonstrated a substantial therapeutic effect. Moreover, by successfully engrafting myoblasts into intact muscle, we demonstrated that even age-related muscle atrophy could be treated by cell therapy as well as muscular diseases, establishing transplantation as a method applicable for human treatment.

We noted two patterns of cell engraftment in skeletal muscle by observing the images of engrafted myoblasts in the muscle tissue. One is seen in myofibers that are newly formed solely by the transplanted myoblasts, which are small cells with central nuclei indicative of myogenesis. These myofibers have bright GFP signals because they are entirely composed of GFP + cells. The other is seen where transplanted cells fuse with existing myofibers. These myofibers show a relatively weak GFP signal because they are composed of GFP + myoblasts and GFP-negative myofibers. The nuclei of the cells are localized just under the plasma membrane, indicating that the cells are fully matured and not regenerated. Both patterns were observed in equal amounts in tissue after transplantation with Matrigel, suggesting that ECM components contribute to both the fusion of myoblasts with each other and with existing myofibers. However, the mechanism by which Matrigel enhanced the myoblast engraftment is unclear. Whether Matrigel provides myoblasts for the space, which is ECM rich and supports myogenesis, or whether fusion is promoted by physical crowding remains to be determined.

Because the engrafting efficiency depends on the degree of stemness of satellite cells (Xie et al., 2021), we note that previous works concluded that undifferentiated cells are singularly effective for skeletal muscle cell transplantation. Satellite cells, known to express high levels of paired transcription factor (Pax7) and low levels of myoblast determination protein 1 (MyoD), do, in fact, engraft preferentially when compared to myoblasts, with their low Pax7 and high MyoD expression (Ikemoto et al., 2007; Sacco et al., 2008). This has led to many groups trying to expand satellite cells while maintaining their stemness to prevent the reduction of graft efficiency (Ito et al., 2016). In notable contrast to these efforts, we instead investigated how myoblasts that have lost their stemness might still be engrafted into skeletal muscle tissue. Myoblasts have the advantage of being more easily cultured *ex vivo* to obtain the large numbers required for cell therapy. We found that ECM components were a key factor for engraftment of transplanted myoblasts and that the niche also played an important role in transplantation efficiency. Our data demonstrate that differentiated myoblasts do not lose engraftment ability, and if the niche is suitable for engraftment, it can contribute to myogenesis. However, the cell transplantation with a high concentration of Matrigel resulted in collagen deposition despite successful cell engraftment. While increasing the number of transplanted cells improved collagen deposition to some extent, it did not completely prevent collagen accumulation, which remains a significant challenge in adapting this approach for regenerative medicine in human application. To address this hurdle, we are exploring the factors contributing to myoblast engraftment among the more than 1,200 proteins included in Matrigel (Hughes et al., 2010). Identifying these key factors could pave the way for developing a more optimized transplantation method for regenerative medicine.

Another limitation is that muscle strength was not increased by myoblast transplantation, even though muscle weight was augmented and GFP + fibers had synapses and capillaries. When 1.0 × 10^6^ myoblasts were transplanted with 2.5 mg/mL Matrigel, both matured GFP + myofibers and immature GFP + myofibers were observed. It may be the case that if these immature myofibers could be matured, muscle strength would increase. One of the reasons why GFP + fibers remained immature in this study is that we used cultured myoblasts directly in the transplantation. This approach could include non-functional cells for myogenesis due to their heterogeneity. Using a Matrigel with reduced amounts of growth factors could also have caused the failure of myofiber maturation. If some factors, such as epidermal growth factor, hepatocyte growth factor, insulin-like growth factor, fibroblast growth factor, or platelet-derived growth factor, were injected along with the myoblasts or delivered after transplantation, mature myofibers would be generated from the transplanted cells (Huey, 2018; Hamaguchi et al., 2023). Physical exercise would also be a valid strategy for maturing myofibers. The muscle contraction associated with exercise promotes myogenesis and muscle hypertrophy (McLeod et al., 2024). Therefore, we need to explore the transplantation method further, including how transplanted cells may be matured.

Recently, many groups have tried to use embryonic stem cells (ES cells) or induced pluripotent stem cells (iPS cells) instead of primary somatic stem cells (Darabi et al., 2012; Goudenege et al., 2012; Kim and Perlingeiro, 2022). ES/iPS cells could be differentiated into myogenic cells by induction of Pax7 or MyoD expression through gene editing (Darabi et al., 2012; Goudenege et al., 2012; Kim and Perlingeiro, 2022); myogenic cells induced from ES/iPS cells, once transplanted, were engrafted into muscle tissue. These cells also indicated higher engraftment efficiency than myoblasts derived from primary cells due to the maintenance of stemness (Boyer et al., 2021); ES/iPS cells could also be expanded *in vitro* before being induced to become myogenic cells (Kim and Perlingeiro, 2022; Boyer et al., 2021). However, ES/iPS cells experience activation of proliferation during the induction process, which increases the risk of cancer cells; preparing cells for transplantation requires a few weeks or months to exclude cancer cells. On the other hand, myoblasts derived from primary somatic stem cells could be expanded *ex vivo* and differentiated without cancer risk. Here, we showed that myoblasts, transplanted with ECM components, could also be effectively engrafted into intact muscle tissue. Moreover, we proved not only that myoblasts engraft morphologically but also that muscle weight could be substantially increased by transplantation. These advantages combine to deliver a breakthrough for cell transplantation as a treatment: myoblasts can be expanded *in vitro* in sufficient numbers for therapeutic applications, and the method does not require muscle injury, making it an ideal option for treating age-related muscle atrophy in humans.

## Data Availability

The original contributions presented in the study are included in the article/Supplementary Material; further inquiries can be directed to the corresponding author.
