# Supplementary material for: Achieving myoblast engraftment into intact skeletal muscle via extracellular matrix
Source: Front Cell Dev Biol. 2025 Jan 14;12:1502332. doi: 10.3389/fcell.2024.1502332 (PMC11772487; doi:10.3389/fcell.2024.1502332)
Supplement: Supplementary file 1 [file DataSheet1.pdf]

Supplementary Material

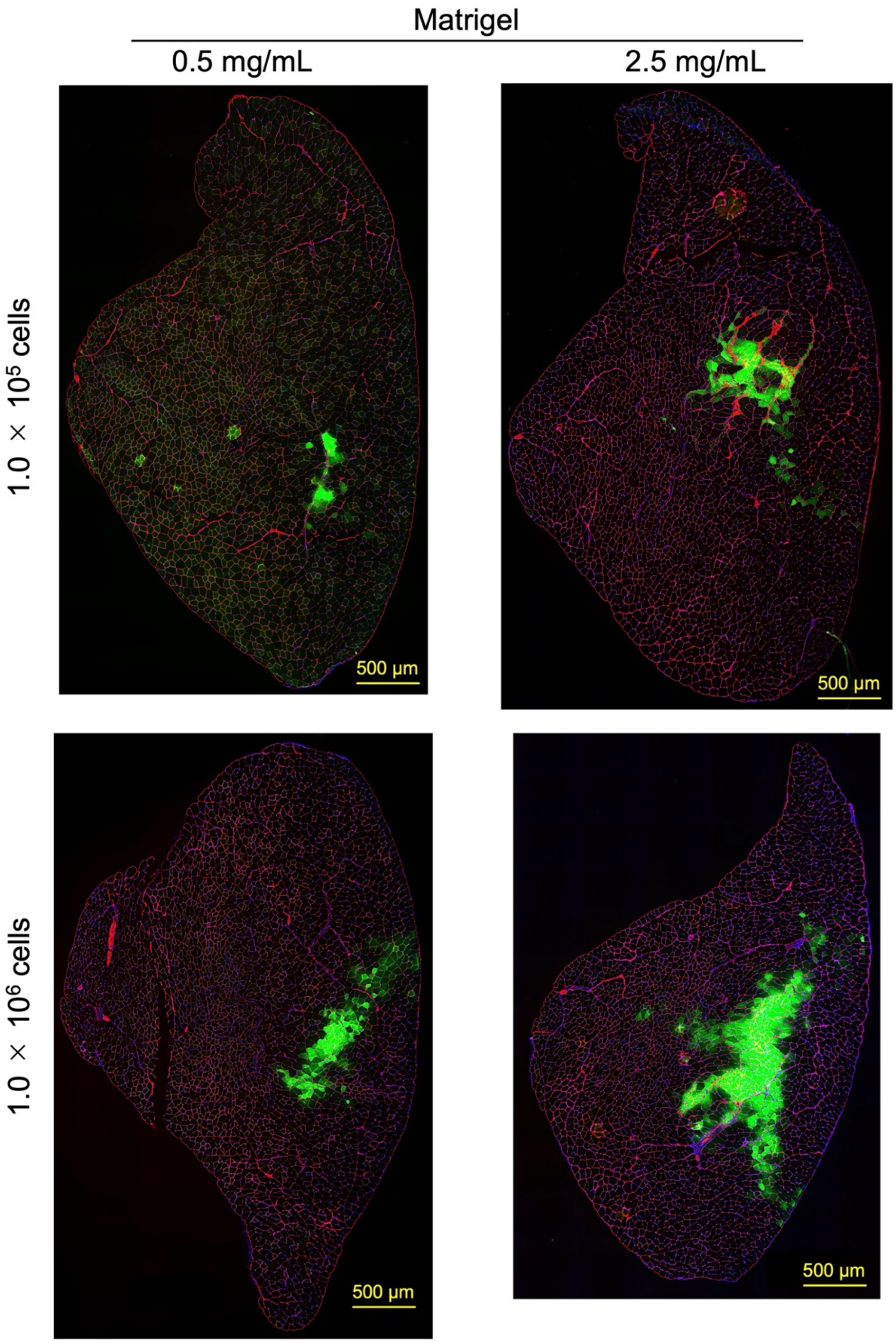

Supplementary Figure 1 Whole TA muscle sections 21 days after myoblast transplantation

The whole transplantation TA sections from the same images shown in Figure 4A. Sections were stained with anti-Laminin-α2 (red) and DAPI (blue) after capturing GFP (green) images. Upper and lower images indicate concentration of Matrigel mixed with the cells. Scale bars are 500 µm.

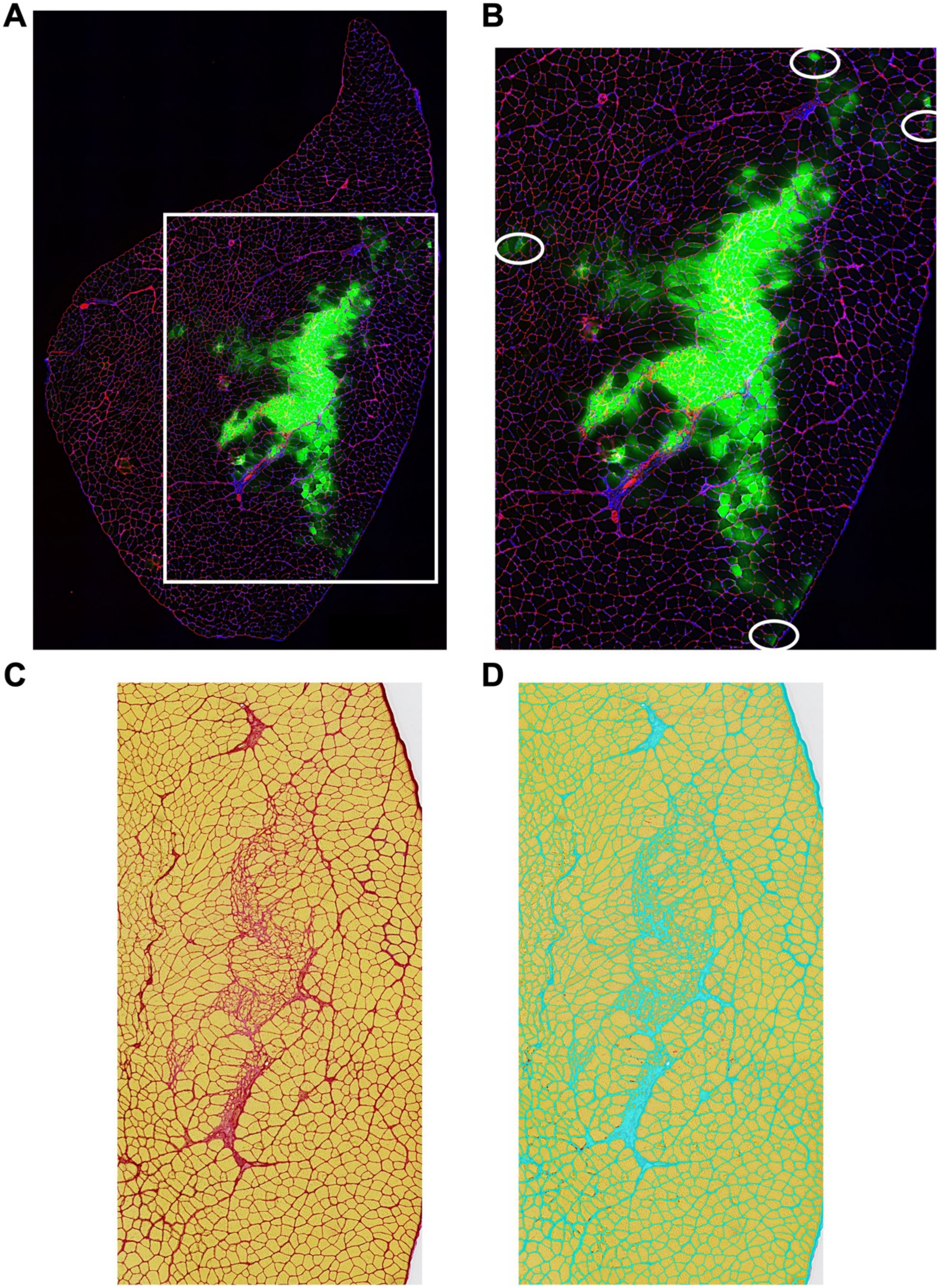
A
B
C
D

Supplementary Figure 2 Definition of the region of interest (ROI) for collagen area analysis

(A) Section of the whole transplanted TA muscle. The colors indicate GFP (green), Laminin-α2 (red), and DAPI (blue). All GFP+ fibers within the section enclosed by white lines.
(B) Rectangular section in (**A**) expanded. The white circles indicate the outermost GFP+ fibers in the section. Note that these circles define the edges of the rectangular region. We define this region as the region of interest (ROI) indicating the area up to which injected myoblasts and Matrigel could infiltrate.
(C) A serial section from the same specimen as in (**A**) was stained with Sirius Red. Myofibers were stained yellow, and collagen was stained red. The same ROI as found in (**B**) was identified in the section.
(D) The collagen area in the ROI was identified as the light blue portion of the image shown. The collagen area in the ROI (%) was calculated using the formula {collagen positive area extracted from ROI (μm^2) / TA area in ROI (μm^2)} × 100 using KEYENCE BZ-X series Image Analysis Software.

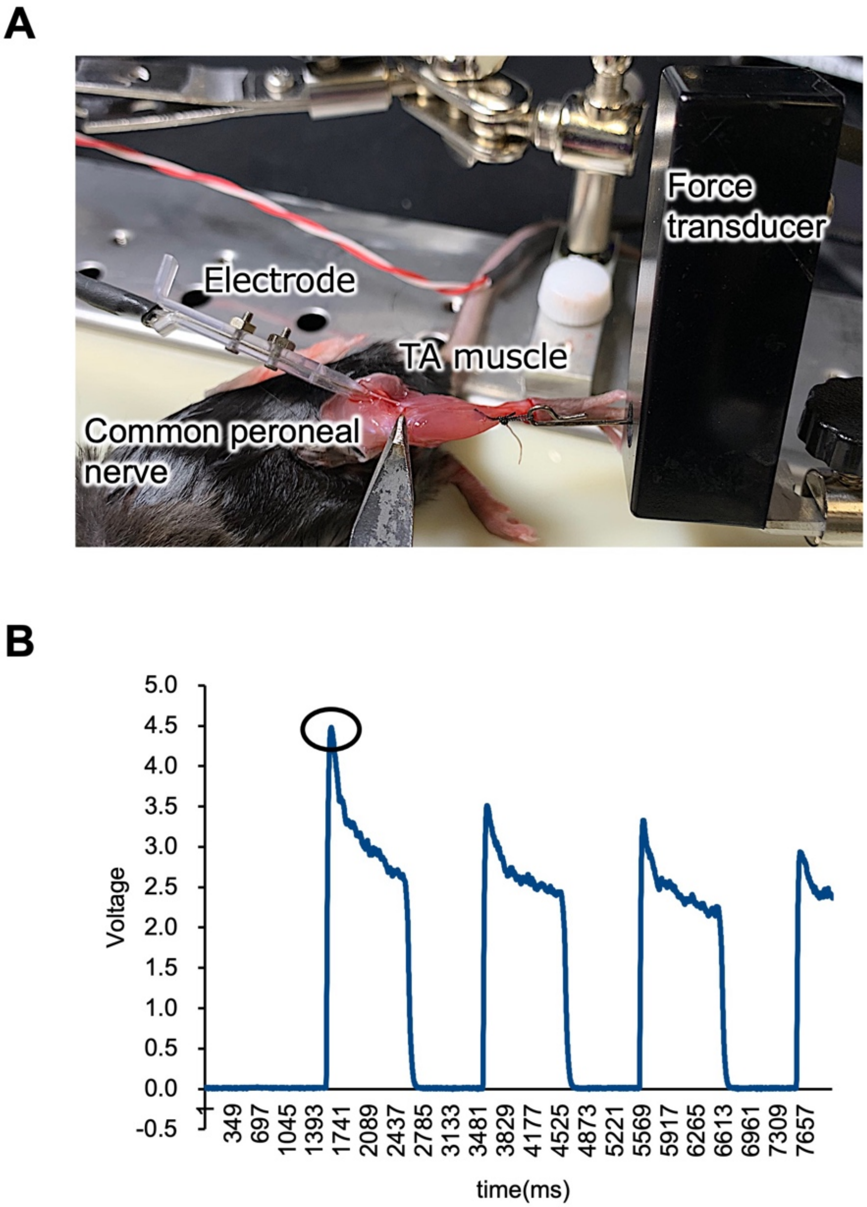

Supplementary Figure 3 TA muscle tension measurement system to evaluate transplanted muscle strength

(A) Under anesthesia, the TA muscle was exposed, and its distal tendon was attached to the force transducer. The electrodes were placed on the common peroneal nerve for electrical stimulation (1.0 voltage pulses, 0.2 ms duration, 250 Hz). The mouse knee was fixed with forceps, and muscle strength was measured in vivo.

(B) The maximal tetanic force was determined from 250 Hz stimulation with 0.2 ms pulses for 10 sec. The maximum voltage in the first contraction (circled in black) was determined as the contraction force. The contraction force is given as a voltage, but is converted to grams using a conversion factor of 10 V to 200 g.

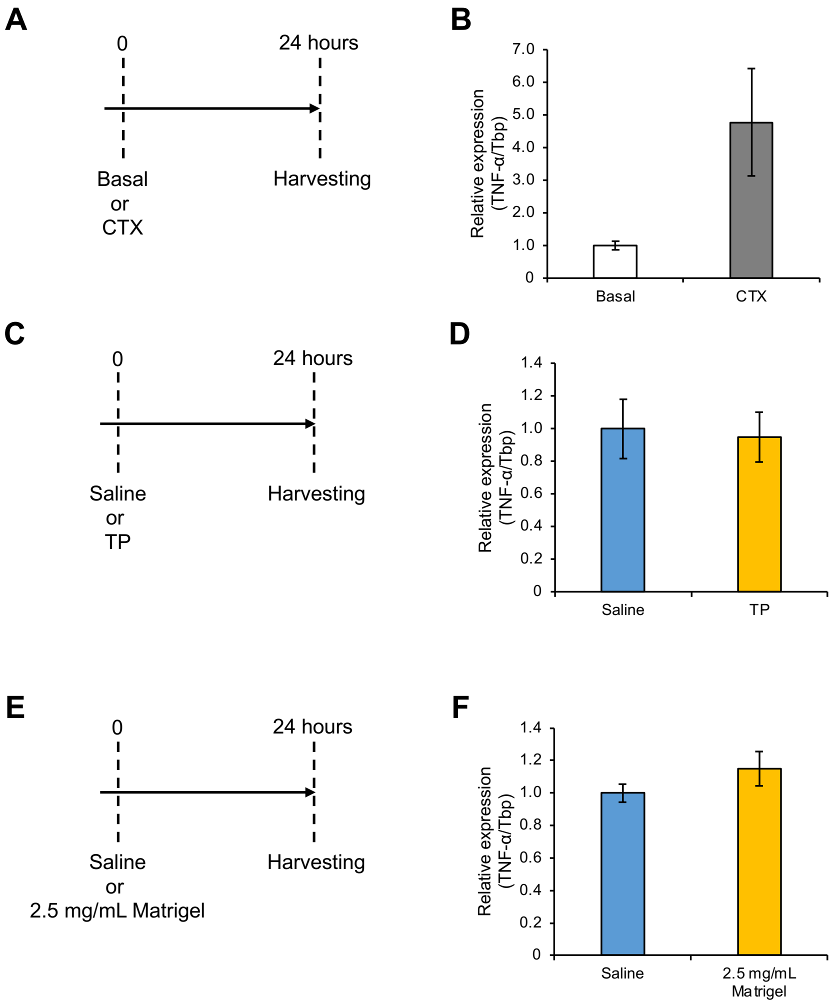

Supplementary Figure 4 Immune responses following muscle injury, myoblast transplantation, and the injection of high concentration of Matrigel

(A) The experimental schedule of muscle injury and analysis. The TA muscle was injured by cardiotoxin (CTX) injection and the contralateral TA muscle basal condition without needle injection. After 24 hours, TA muscles were removed and analyzed.

(B) The mRNA expression on TNF-α was measured by qPCR. Data are shown as the mean ± s.e.m. (n= 2-3).

(C) The experimental schedule of myoblast transplantation and analysis. The saline was injected into TA muscle and 1.0×10^5 myoblasts mixed with 0.5 mg/mL Matrigel were transplanted into the contralateral TA muscle, shown as TP condition. After 24 hours, TA muscles were removed and analyzed.

(D) The mRNA expression on TNF-α was measured by qPCR. Data are shown as the mean ± s.e.m. (n= 5). Statistical significance was determined by Student's t-test, but there was not significant.

(E) The experimental schedule of the injection of high concentration Matrigel. The saline was injected into TA muscle and 2.5 mg/mL Matrigel were transplanted into the contralateral TA muscle. After 24 hours, TA muscles were removed and analyzed.

(F) The mRNA expression on TNF-α was measured by qPCR. Data are shown as the mean ± s.e.m. (n= 4). Statistical significance was determined by Student's t-test, but there was not significant.

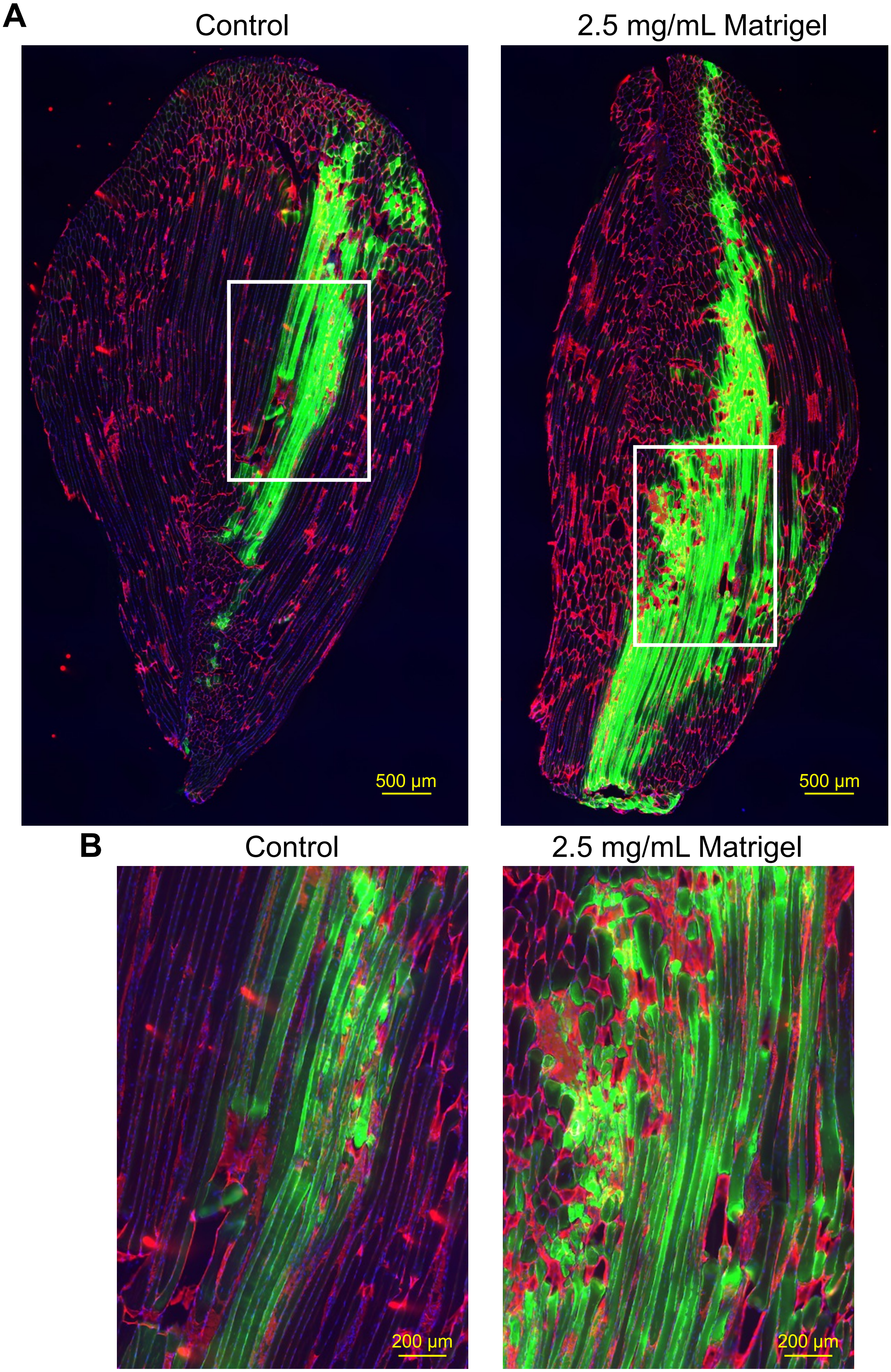
A
Control
2.5 mg/mL Matrigel
500 μm
500 μm
B
Control
2.5 mg/mL Matrigel
200 μm
200 μm

Supplementary Figure 5 Longitudinal cryosection of transplanted TA muscle

(A) The longitudinal cryosection of TA muscles 21days after myoblast transplantation. Sections were stained with anti-Laminin-α2 (red) and DAPI (blue) after capturing GFP (green) images. Left image indicates 1.0×10^6 myoblasts transplanted into intact TA muscle without Matrigel, shown as Control condition, and right image indicates myoblasts mixed with 2.5 mg/mL Matrigel transplanted. Scale bars are 500 µm.

(B) The expanded images from the white rectangle of panel **(A)**. Scale bars are 200 µm.

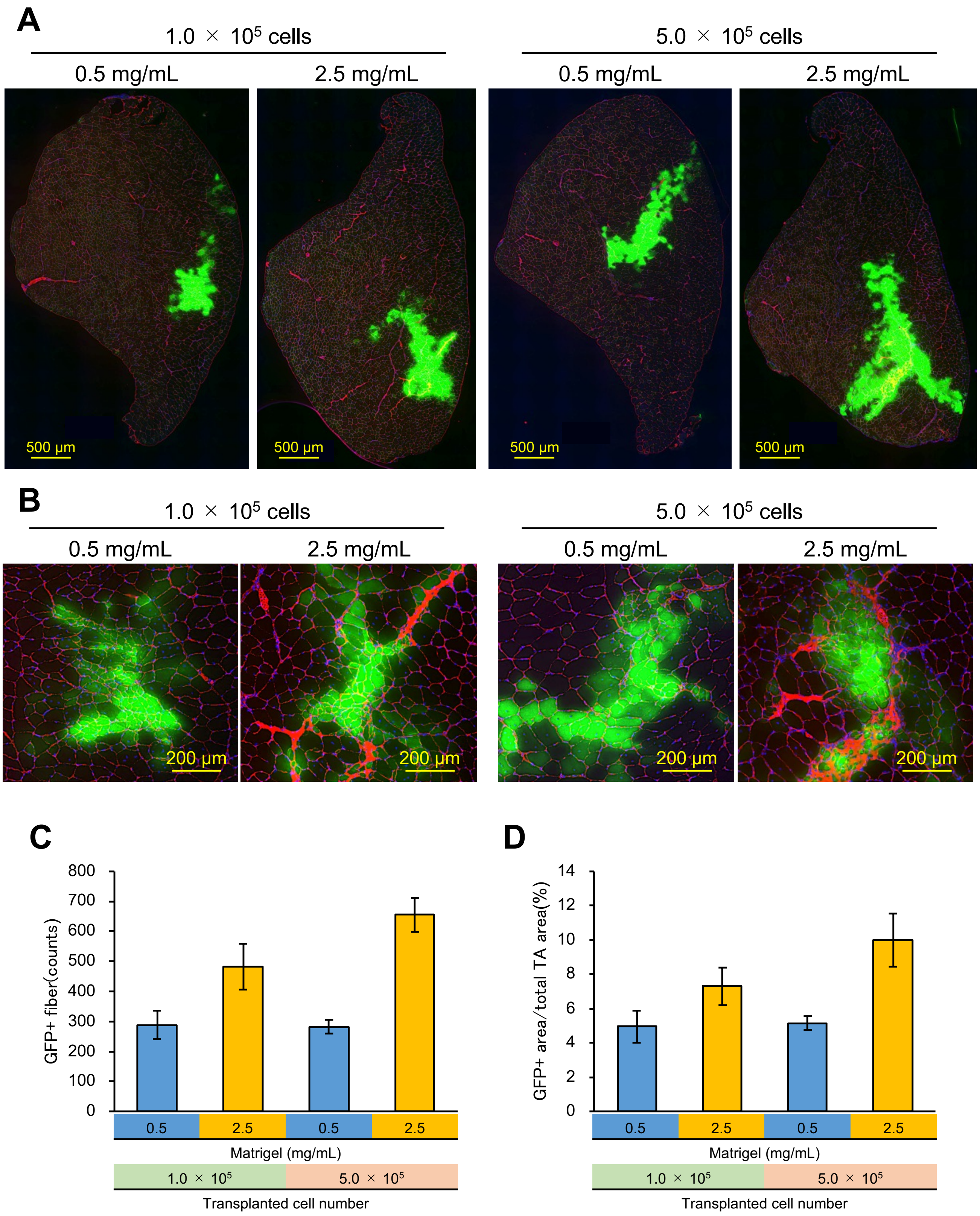

Supplementary Figure 6 Myoblast engraftment into intact skeletal muscle 6 weeks after transplantation

(A) 1.0×10^5 or 5.0×10^5 myoblasts were mixed with 0.5 or 2.5 mg/mL Matrigel, then transplanted into intact TA muscle of WT mice. Frozen sections were prepared from TA muscle 6 weeks after myoblast transplantation. After capturing GFP (green) images, the sections were stained with anti-Laminin-α2 (red) and DAPI (blue). Scale bars are 500 µm.

(B) The expanded images from panel **(A)**. Scale bars are 200 µm.

(C) The number of GFP positive fibers was counted from the images in panel **(A)**. Data are shown as the mean ± s.e.m. (n= 3-4).

(D) GFP+ area was also measured from the images in panel **(A)**. Data are shown as the mean ± s.e.m. (n= 3-4).

A

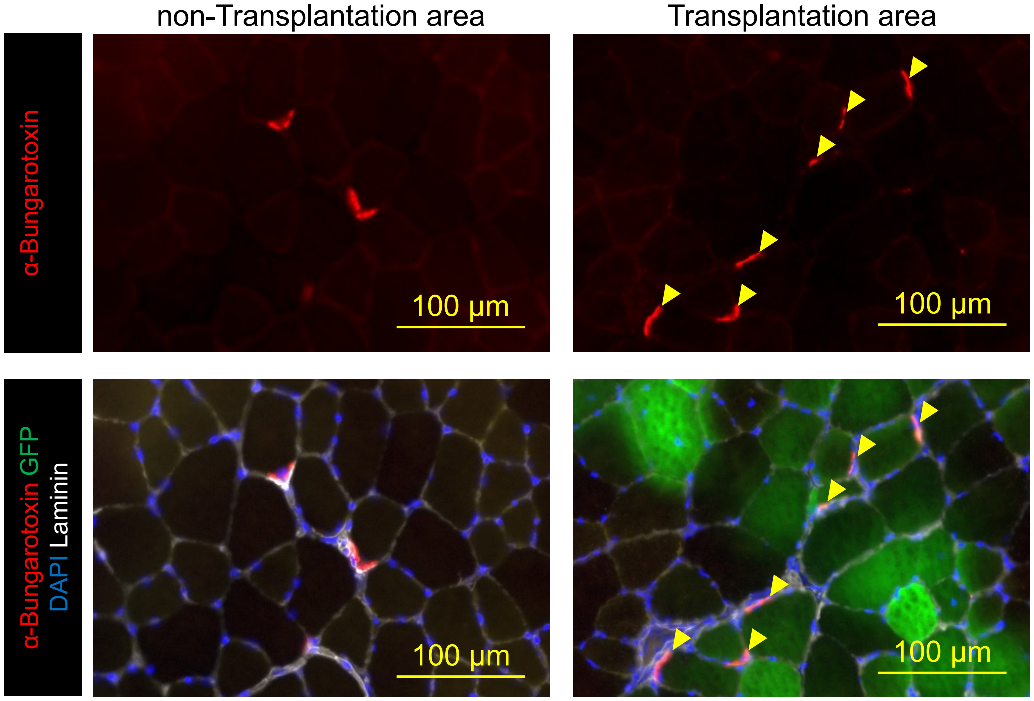

B

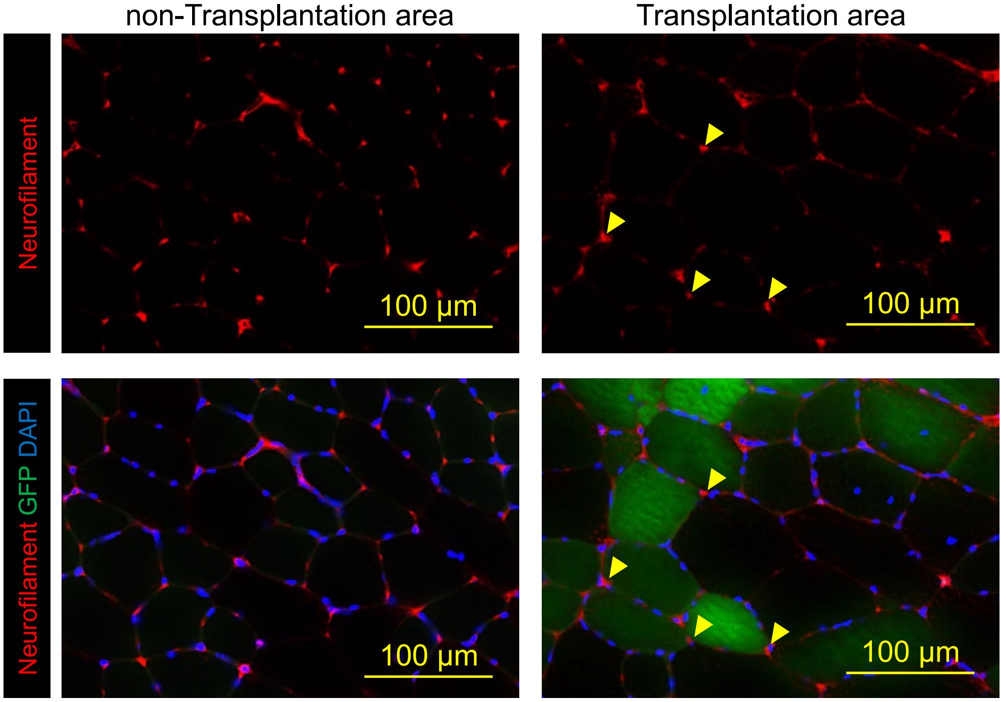

Supplementary Figure 7 The synapse structures in transplanted skeletal muscle tissue

(A, B) The Frozen sections were prepared from TA muscle 21 days after myoblast transplantation mixed with 2.5 mg/mL Matrigel. The images of each panel were captured in the same section, which non-Transplantation area indicated there were not any GFP+ fibers and Transplantation area included GFP+ fibers.

(A) Transplanted muscle section was stained with α-Bungarotoxin (red), GFP (green), DAPI (blue), and anti-Laminin-α2 (white). The yellow arrow heads indicate that α-Bungarotoxin and GFP are merged. Scale bars are 100 µm.

(B) Transplanted muscle section was stained with anti-Neurofilament (red), GFP (green), and DAPI (blue). The yellow arrow heads indicate that Neurofilament and GFP are merged. Scale bars are 100 µm.

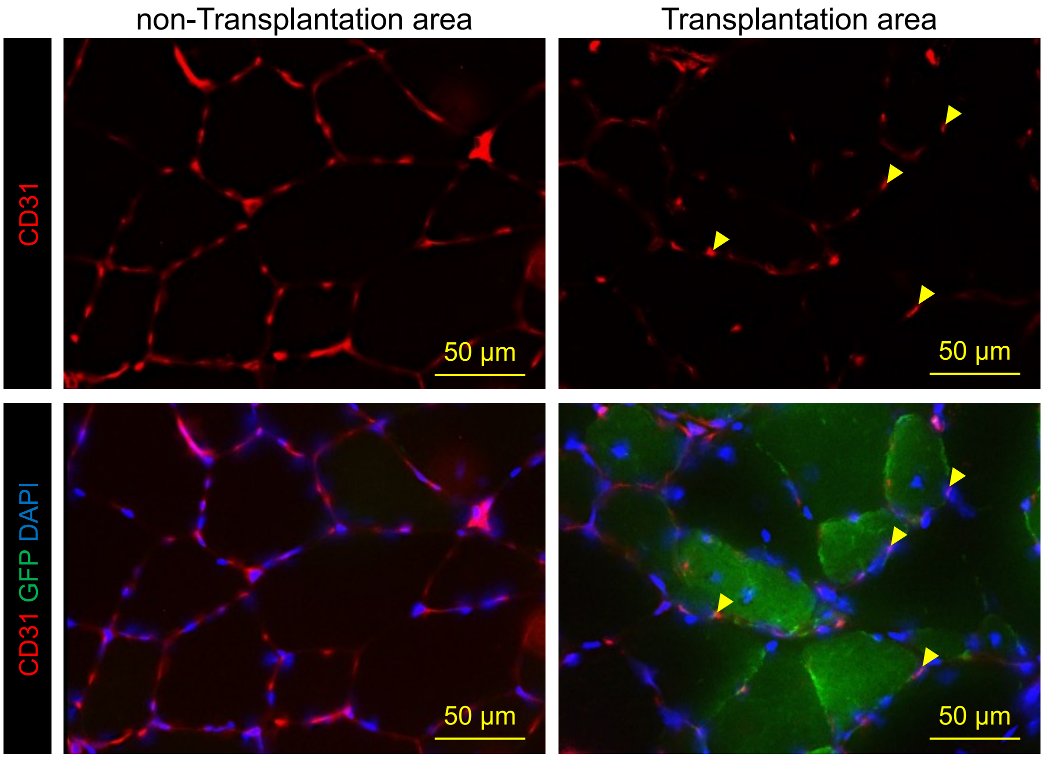

Supplementary Figure 8 The CD31 localization in transplanted skeletal muscle tissue

Transplanted muscle section was stained with CD31 (red), GFP (green), and DAPI (blue). The yellow arrow heads indicate that CD31 and GFP are merged. Scale bars are 50 µm.
